# Relationship between Polycyclic Aromatic Hydrocarbon–DNA Adducts, Environmental Tobacco Smoke, and Child Development in the World Trade Center Cohort

**DOI:** 10.1289/ehp.10144

**Published:** 2007-05-31

**Authors:** Frederica P. Perera, Deliang Tang, Virginia Rauh, Yi Hsuan Tu, Wei Yann Tsai, Mark Becker, Janet L. Stein, Jeffrey King, Giuseppe Del Priore, Sally Ann Lederman

**Affiliations:** 1 Columbia Center for Children’s Environmental Health, Mailman School of Public Health, Columbia University, New York, New York, USA; 2 Department of Statistics, National Cheng Kung University, Taiwan, Republic of China; 3 Center for International Earth Science Information Network of the Columbia University Earth Institute, Palisades, New York, USA; 4 Department of Obstetrics and Gynecology, Beth Israel Medical Center, New York, New York, USA; 5 Department of Obstetrics and Gynecology at St. Vincent’s Medical Center, New York, New York, USA; 6 Department of Obstetrics and Gynecology at New York University Downtown Hospital, New York, New York, USA

**Keywords:** child development, DNA adducts, ETS, *in utero*, PAHs, World Trade Center

## Abstract

**Background:**

Polycyclic aromatic hydrocarbons (PAHs), including benzo[*a*]pyrene (BaP), are air pollutants released by the World Trade Center (WTC) fires and urban combustion sources. BaP–DNA adducts provide a measure of PAH-specific genetic damage, which has been associated with increased risk of adverse birth outcomes and cancer. We previously reported that levels of BaP–DNA adducts in maternal and umbilical cord blood obtained at delivery were elevated among subjects who had resided within 1 mile of the WTC site during the month after 9/11; and that elevated blood adducts in combination with *in utero* exposure to environmental tobacco smoke (ETS) were significantly associated with decreased fetal growth.

**Objective:**

Our aim was to assess possible effects of prenatal exposure to WTC pollutants on child development.

**Methods:**

After 11 September 2001, we enrolled a cohort of nonsmoking pregnant women who delivered at three lower Manhattan hospitals. We have followed a subset of children through their third birthdays and measured cognitive and motor development using the Bayley-II Scales of Child Development (BSID-II).

**Results:**

In multivariate analyses, we found a significant interaction between cord blood adducts and *in utero* exposure to ETS on mental development index score at 3 years of age (*p* = 0.02, *n* = 98) whereas neither adducts nor ETS alone was a significant predictor of (BSID-II) cognitive development.

**Conclusion:**

Although limited by small numbers, these results suggest that exposure to elevated levels of PAHs in conjunction with prenatal ETS exposure may have contributed to a modest reduction in cognitive development among cohort children.

In this research we focused on possible neurodevelopmental risks of prenatal exposure to pollutants emitted by the World Trade Center (WTC) fires because of prior experimental and human evidence that some of the pollutants can affect the developing brain and adversely affect cognitive development (Perera et al. 2006; Wormley et al. 2004). Of interest were possible interactions between polycyclic aromatic hydrocarbons (PAHs) (measured here by benzo[*a*]pyrene [BaP]–DNA adducts in cord blood) and prenatal environmental tobacco smoke (ETS) exposure, as was previously seen for fetal growth in this same cohort (Perera et al. 2005). The destruction and combustion of the WTC towers released a complex mixture of toxicants into the New York City environment on and after 11 September 2001 (Lioy et al. 2002; McGee et al. 2003; Offenberg et al. 2003). These included pollutants with neurodevelopmental toxicity and carcinogens such as PAHs, poly-chlorinated biphenyls (PCBs), polychlorinated dibenzodioxins, polychlorinated dibenzofurans, polybrominated diphenyl ethers, and various metals (Chen and Thurston 2002; Jeffrey et al. 2003; Lioy et al. 2002; McKinney et al. 2002; Offenberg et al. 2003). The WTC plume contained high levels of PAHs that spiked at a measurement site 1.8 km (1.1 mile) northeast of the WTC site several times in September and October 2001, with a peak on 3 October during an inversion that brought smoke back to ground level (Service 2003). PAHs are also common pollutants in urban air from fossil fuel combustion by motor vehicles, residential heating units, power plants, and industrial activities (Bostrom et al. 2002) and are present in tobacco smoke and in grilled or broiled food (International Agency for Research on Cancer 1983; U.S. Environmental Protection Agency 1990). Thus, during the weeks and months after 11 September 2001, the WTC fires added to an ongoing background exposure to airborne PAHs. Several PAHs, including BaP (Bapat et al. 1994), are known human mutagens, carcinogens, and/or developmental toxicants. BaP is widely used as a representative PAH because concentrations of individual PAHs in the urban setting are highly intercorrelated (Perera et al. 2003). Therefore, we have used BaP–DNA adducts as a proxy for PAH–DNA adducts. Because they reflect individual variation in exposure, absorption, metabolic activation, and DNA repair, the adducts in white blood cells provide a biologic dosimeter and marker of potential risk (Bartsch and Hietanen 1996; Veglia et al. 2003). DNA adducts have an estimated half-life of 3–4 months (Mooney et al. 1995). Thus, considering that the main exposure to WTC-related PAHs occurred between 11 September 2001 and 11 November 2001 while the fires were ongoing, adduct measurements in blood samples collected between December 2001 and June 2002 would partly reflect that exposure.

Here we focus on possible risks of the WTC disaster to children of women who were pregnant at that time because of evidence that the fetus is more sensitive than the adult to a range of pollutants including PAHs (National Research Council 1993; Perera et al. 2004; Whyatt et al. 2001). For example, compared with their mothers, newborns sampled at delivery have more genetic damage (in the form of DNA adducts) per estimated unit of exposure to PAHs; and they demonstrate slower clearance of various toxicants (National Research Council 1993; Perera et al. 2004; Whyatt et al. 2001).

Our previous findings in this cohort raised some concern about possible neurodevelopmetal effects of PAHs from the WTC event. First, we found that PAH–DNA adducts were highest among those mothers and newborns in the cohort who had at some time in the 4 weeks after 11 September 2001 resided within 1 mile of the WTC site; adduct levels were intermediate among those who worked but did not live within 1 mile of the WTC during this time period; and they were lowest among those who neither worked nor lived within this radius (Perera et al. 2005). Second, we observed a significant interaction between cord adducts treated as a continuous variable and maternal prenatal ETS exposure on birth weight (*p* = 0.03) and head circumference (*p* = 0.04) (Perera et al. 2005). These findings suggested the need for follow-up of the children, because other research has found associations between reduced birth weight, even in the normal range, and health and developmental problems (Barker 1996; Dietz 1994; Matte et al. 2001; Rice and Barone 2000; Richards et al. 2002). Therefore, we examined the relationships between PAH–DNA adducts measured in umbilical cord blood, prenatal ETS exposure, and neurodevelopmental outcomes at 3 years of age in a subset of the WTC cohort who have been prospectively followed through that time point.

## Materials and Methods

### Recruitment, data collection, and geocoding

**Figure 1:**
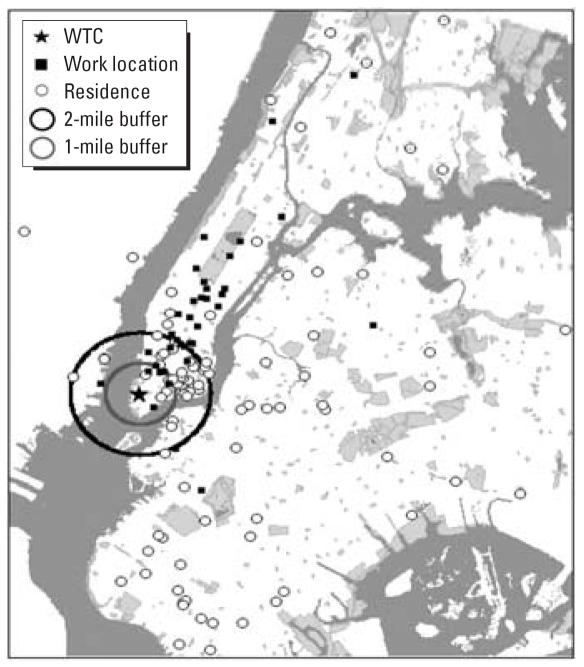
Location of residences and workplaces of subjects included in the current analysis of adducts ETS, and Mental and Psychomotor Development Indexes. Map shows the 1- and 2-mile radius from the WTC site. Map provided by the Center for International Earth Science Information Network (CIESIN), Columbia University. Prepared by M.B.

This study is a project within the Columbia Center for Children’s Environmental Health (CCCEH; www.ccceh.org). The study methods have been described previously (Lederman et al. 2004). Patients were enrolled at Beth Israel, St. Vincent’s, and New York University Downtown Hospitals and St. Vincent’s affiliated Elizabeth Seton Childbearing Center, selected because of their close proximity to the WTC site. Singleton pregnant women were enrolled at the time of labor. Eligible women were between 18 and 39 years old, had not smoked during pregnancy, and reported no diabetes, hypertension, HIV infection or AIDS, or use of illegal drugs in the preceding year. Enrollment began on 12 December 2001, as soon as institutional review board approval was obtained, and ended on 26 June 2002. The enrolled women had been pregnant on 11 September or, in two cases, became pregnant during the month following. Women were briefly screened for eligibility, recruited, enrolled, consented before delivery, and interviewed after delivery by bilingual interviewers in their preferred or native language (English, Spanish, or Chinese). Of the women initially screened for eligibility, 369 women were eligible and gave consent for participation; 329 contributed at least one blood sample (286 cord, 212 maternal samples), medical record information, and a postpartum interview, all of which were required for full enrollment in the study. Information on the pregnancy, delivery, and birth outcomes was collected from the medical records of the mother and newborn (Lederman et al. 2004). A 30- to 45-min interview was administered to each mother after delivery to elicit information on demographics; reproductive history; background environmental exposures including ETS (number of smoking household members and regular visitors to the home), dietary PAH exposure via grilled, smoked, and barbecued foods; and the location of the woman’s residences and workplaces during each of the 4 weeks after 11 September 2001 (Lederman et al. 2004). Similar questionnaires were administered by interview with the mother when the children were 1, 2, and 3 years of age. Residential and work addresses were geocoded at the Center for International Earth Science Information Network of Columbia University’s Earth Institute; and the geocoded linear distance from the WTC site was computed for each residence and work site (GIS software, including ArcGIS 8.3 and the StreetMap 2003 extension; Environmental Systems Research Institute, Redlands, CA). Figure 1 shows the location of residences and workplaces of subjects included in the current analysis.

### Blood collection and adduct analysis

Umbilical cord blood (mean 30.7 mL) was collected at delivery and maternal blood (30–35 mL) generally on the first day after delivery. Samples were transported to the CCCEH Molecular Epidemiology Laboratory within several hours of collection. The buffy coat, packed red blood cells, and plasma were separated and stored at −70°C.

BaP–DNA adducts in extracted white blood cell DNA were analyzed using the high-performance chromatrography (HPLC)/ fluorescence method of Alexandrov et al. (1992), which detects BaP tetraols (Alexandrov et al. 1992; Rojas et al. 1994); the method was modified as described previously (Perera et al. 2004). Briefly, we used about 100 μg of DNA for each analysis. Many precautions were taken to avoid the presence of fluorescent contaminants. DNA samples were dissolved in 0.1 N HCl, and hydrolyzed at 90°C for 6 hr. We analyzed the resulting solution in a Shimadzu HPLC system with RF-10Axl spectrofluorometric detector. The Shimadzu S IL-10A automatic sample injector (Shimadzu, Kyoto, Japan) was used to minimize any batch effect. We calculated the tetraol concentrations by comparing the samples analyzed with an external calibration curve, generated from the fluorescence peak of a known amount of authentic benzo[*a*]pyrene diol epoxide (BPDE) tetraol standard, every time a set of samples was analyzed. Calibration was carried out with DNA from calf thymus alone (background) and spiked with 2, 4, and 8 pg anti-BPDE tetraol. These standard solutions were then treated in the same way as the tested samples. The correlation coefficient was 0.98, and the mean coefficient of variation for analyses repeated on different days was 12%. The detection threshold of BPDE tetraols [r-7,c-10,t-8,t-9-tetrahydroxy-7,8,9,10-tetrahydrobenzo[*a*]pyrene (BaP tetraol I-1) and r-7,t-9,t-10,t-8-tetrahydroxy-7,8,9,10-tetrahydrobenzo[*a*]pyrene (BaP tetraol I-2)] was 0.25 pg (signal-to-noise ratio > 3) so that, in the present study, with 100 μg of DNA, this assay could detect 0.25 adducts per 10^8^ nucleotides. Assays were performed on all samples that were of adequate quantity and quality for analysis.

### Description of the sample

The parent study population was diverse, reflecting the mixed residential and commercial nature of lower Manhattan and the broader area served by the delivery hospitals (Lederman et al. 2004). Blood donation has been a cultural taboo in China (Gill et al. 2002), and many of the enrolled Chinese women provided only a cord blood sample. As previously reported, the subset of 203 subjects with newborn cord blood adduct data did not differ from those without cord adduct measurements with respect to maternal age, income, education, ethnicity, gestational duration, and prenatal exposure to ETS (Perera et al. 2005). The subset in the present report included the 98 children with data on cord adducts, 3-year Bayley assessments [Bayley Scales of Infant Development, 2nd ed.; BSID-II (Bayley 1993)], and other demographic and exposure variables required for the regression analysis. One hundred five children with cord adduct data were excluded because they lacked Mental Development Index (MDI) (*n* = 89) and/or complete demographic/ exposure data that were required for the analysis. As shown in Table 1, the group included in the analysis was similar in maternal age, income, ethnicity, and gestational duration to the group of 105 children who were not included. However, a higher proportion of the included group lived within 2 miles of the WTC, compared with the group not included.

### Developmental testing

Not all mothers had agreed at delivery to have their children followed after birth (some of the Chinese children were scheduled to return to China), and not all children were available at all ages. Women were contacted and reinterviewed at approximately 6-month intervals after delivery until their children were 3 years of age. At the annual time points, the children were invited to the CCCEH offices for measurement of weight, height, and head circumference and for assessment of cognitive and psychomotor development using the BSID-II (Bayley 1993) through 3 years of age. The BSID-II was administered by trained research workers when children were 3 years ± 6 months of age. Assessments were done using validated test methods. In some cases, the assessments were done in Chinese by bilingual research staff. The BSID-II testing process accounts for age at the time of testing. The BSID-II is widely used and norm-referenced, can be used to diagnose developmental delay, and has been shown to be sensitive to the effects of low-level intrauterine exposure to lead (Bellinger et al. 1987) and PAHs (Perera et al. 2006). The test provides a Developmental Quotient (raw score/ chronological age), which generates a continuous MDI and a corresponding Psychomotor Development Index (PDI). The stability of cognitive assessments during the first few years of life is limited, but the predictive power increases as the child ages. When administered at 3 years of age, the BSID-II demonstrates moderate predictive power for subsequent intelligence and school performance, and is clinically useful for identifying children performing in the subnormal range (Bayley 1993; Burchinal et al. 2000; Sternberg et al. 2001). Children are classified based on their test score, as normal (> 85), moderately delayed (≥ 70 and ≤ 85), or severely delayed (≤ 70).

Maternal intelligence was measured by the Test of Nonverbal Intelligence–Second Edition (TONI-2) (Brown et al. 1990), a 15-min, language-free measure of general intelligence, relatively stable and free of cultural bias. The test was administered when the child was brought in for assessment. Mothers were retested with an alternate test version, if they came for a subsequent visit. The mean of the two values was then used as their score.

### Statistical methods

The present analyses are based on cord blood adducts. Adduct levels were treated in the analyses both continuously and categorically (detectable or nondetectable). As in prior studies (Perera et al. 2005), samples with nondetectable PAH–DNA adducts were given a value of one-half the limit of detection (LOD/2 = 0.125). DNA adducts were right-skewed and were therefore log-transformed to meet the normality assumption of the linear regression model. However, means are presented in arithmetic scale for ease of interpretation. MDI and PDI were normally distributed (*p* = 0.32 for both MDI and PDI by the Kolmogorov-Smirnov test) and were not log-transformed. A residual plot showed that the linear model that we fit was reasonable.

Cord adducts and ETS were not correlated (*r* = 0.01, *p* = 0.92) by Spearman rank correlation test. We used multiple linear regression to assess the effect of continuous (log) PAH–DNA adducts, independently and combined with ETS, on child development as indicated by MDI and PDI scores on the BSID-II test at 36 months of age. In separate models, we treated adducts as dichotomous (nondetectable, detectable). To control for potential confounding, we tested a number of sociodemographic and exposure variables for their association with MDI and PDI; these were included in the analysis if they were univariately associated with the outcome at *p* < 0.1. Sex of child, gestational age, ethnicity, maternal age, income, and education were removed from the models because they did not meet this criterion. Material hardship (defined in Table 1), marital status, and maternal intelligence (TONI) were significant at the level of 0.1 and thus were included in the model.

In addition, to determine whether the exposures were related to the likelihood of developmental delay, we used logistic regression to estimate associations between the independent variables (log cord blood adducts and prenatal ETS exposure) and the dichotomous outcome variable (developmental delay/no developmental delay). Separate models were run using moderate delay/no delay or severe delay/no delay as the outcome variables.

We also adjusted for the effect of postnatal exposure to ETS in the multivariate model. The presence or absence of smokers in the home (household members or regular visitors) through 3 years of age was used to define postnatal exposure to ETS (yes or no). Prenatal and postnatal exposure to ETS were significantly correlated (*r* = 0.45, *p* = <0.0001, Pearson’s correlation). We evaluated possible confounding by postnatal ETS by including postnatal ETS (exposure to household ETS in years 1–3, yes/no) in the models.

We assessed potential mediation by birth weight of the association between PAH–DNA adducts and ETS and MDI by including birth weight—a fetal growth parameter previously shown to be affected by these exposures in the cohort—in the models. If the estimate of the PAH effect on neurodevelopment were attenuated with inclusion of the fetal growth parameter, mediation would be considered to be present.

Finally, as in prior analyses (Lederman et al. 2004), we tested in regression models whether proximity of residence to the WTC site (< 2 miles vs. > 2 miles) during the 4 weeks after the event was a significant predictor of neurodevelopmental outcomes. As shown in Table 1, 34 of the 98 (35%) mothers included in the analysis lived within 2 miles of the WTC in the month after 9/11.

All statistical analyses were carried out using SPSS software (version 14; SPSS Inc., Chicago, IL).

## Results

Demographic and exposure characteristics for the 98 children included in the present analysis are shown in Table 1. The average age of mothers was 30 years; the predominant ethnic/racial group was white (40.8%), followed by Asian (34.7%), and black (13.3%). Seventy percent of the mothers had a greater than high school education, and a quarter were exposed to ETS in the home.

**Figure 2:**
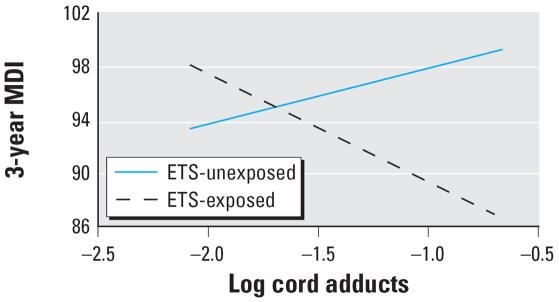
Results of the interaction model showing the relationship between adducts and MDI in the ETS-exposed and ETS-unexposed groups. The relation between MDI and adducts in the ETS-exposed group was of borderline significance (β = −8.07, *p* = 0.07). The relation between MDI and adducts in the ETS-unexposed group was not significant (β = 4.18, *p* = 0.11). The 95% CI for the β for the ETS-exposed group is −17.10 to 0.96; the 95% CI of the β for the ETS-unexposed group is −0.95 to 9.31.

Multiple linear regression analyses showed no significant main effects of cord adducts (*p* = 0.61) or ETS exposure (*p* = 0.37) on MDI or PDI at 3 years of age. However, as shown in Table 2 and Figure 2, there was a significant interaction between adducts treated as a continuous variable and ETS on MDI (β = −12.25*, p* = 0.02). Table 2 and Figure 2 present βs and 95% confidence intervals (CIs) derived from the multiple linear regression model using the continuous measure of adducts. Removing two women who had preterm deliveries (≤ 258 days) did not affect the results (for continuous adducts × ETS, β = −12.71, *p* = 0.02). We estimated that among ETS-exposed subjects, a doubling of PAH–DNA adducts within the observed range corresponds to an average 6% reduction in MDI. The interaction between adducts and prenatal ETS exposure on MDI remained significant after including postnatal exposure to ETS in the multiple linear regression model (β = 11.96, *p* = 0.027).

The effect on MDI of the interaction between ETS and adducts treated as a categorical variable (detectable/nondetectable) was similar to that based on the continuous adduct variable, but was of borderline significance (β = −9.56, *p* = 0.06). Sixty-one children had detectable adducts in cord blood, and 37 children had nondetectable cord adducts. Among the children with detectable adducts, 3-year MDI was lower in the ETS-exposed group than in the ETS-unexposed group (*p* = 0.06). In contrast, among the children with non-detectable adducts, 3-year MDI in the ETS-exposed group did not differ significantly from that in the ETS-unexposed group (*p* = 0.34).

Multiple linear regression analyses did not show effects of adducts and ETS on PDI at 3 years of age.

Seventeen children had MDI scores indicating developmental delay (MDI score ≤ 85) at 3 years of age; and 16 had scores indicating psychomotor delay (PDI score ≤ 85). By logistic regression, there were no significant effects of adducts, ETS, or their interaction on developmental delay (MDI or PDI score ≤ 85) at 3 years of age.

The impact of PAH–DNA adducts and ETS on 3-year mental development does not appear to be mediated by birth weight: Inclusion of birth weight in the model did not attenuate the relationship between adducts, ETS, and MDI (β for adduct × ETS = −12.16, *p* = 0.03)). Proximity of residence to the WTC site was not significantly associated with the neurodevelopmental outcomes (all βs were *p* > 0.05).

## Discussion

The observed interaction between PAH–DNA adducts and prenatal exposure to ETS in this WTC cohort is consistent with the previous finding that birth weight and head circumference were significantly reduced in newborns with the combined exposures. As noted above, reduced fetal growth has been linked to increased risk of developmental problems. The present finding of a decrement in MDI associated with cord blood adduct concentrations among ETS-exposed children is also consistent with the observation in the northern Manhattan/South Bronx cohort that elevated prenatal exposure to airborne PAHs was associated with a reduction in MDI score at 3 years of age (Perera et al. 2006). The effect of increasing PAH–DNA adduct levels among children exposed prenatally to ETS was, however, modest (β = −8.07, *p* = 0.07). In light of the prior finding that adducts were related to proximity of residence to the WTC site during the month after 11 September 2001, these data suggest that, in conjunction with prenatal exposure to ETS, *in utero* exposure to PAHs emitted by the WTC fires during the weeks after 9/11 may have contributed to modest reductions in cognitive development test scores at age 3.

The observed combined effect of PAHs and ETS on child development is biologically plausible. Mechanisms by which *in utero* exposure to PAHs/ PAH–DNA adducts in combination with ETS can affect brain development include antiestrogenic effects (Bui et al. 1986), binding to the human aryl hydrocarbon receptor to induce P450 enzymes (Manchester et al. 1987), DNA damage resulting in activation of apoptotic pathways (Meyn 1995; Nicol et al. 1995; Wood and Youle 1995), or interference with transcription, DNA replication, or protein synthesis (Bostrom et al. 2002). ETS is a complex mixture of > 4,000 chemicals, including PAHs and carbon monoxide (Leikauf et al. 1995). Prenatal exposure to tobacco smoke has been associated previously with deficits in birth weight and birth length (Janerich et al. 1990; Martinez et al. 1994; Schuster-Kolbe and Ludwig 1994; Sexton et al. 1990) and adverse effects on cognitive development (Rauh et al. 2004). In the present study, the impact of PAH–DNA adducts and ETS on 3-year mental development does not appear to be mediated by the effect of these exposures on birth weight.

A major strength of the present study is the prepartum enrollment of exposed and unexposed women from a common clinical population. All women delivered at one of three lower Manhattan hospitals during the same time period. In addition, enrollment occurred before delivery and before the outcome of the pregnancy was known, so that women’s decision to participate was not influenced by their knowledge of birth outcome. Recruitment procedures ensured a wide range of race/ethnicity, income, education, and other characteristics, improving the generalizability of our findings. In addition, the subjects were geographically well dispersed during pregnancy with respect to the WTC site, providing a basis for comparing subjects with differential exposure.

A limitation of the study is the small number of subjects and the fact that, because of the time required to obtain institutional review board approval from the participating hospitals, recruitment did not begin until December 2001. The effects of pollutant exposure during the third trimester could not be examined because such women would already have delivered before the beginning of this study. In addition, given the time lapse of 1–7 months between exposure to WTC air pollutants (which occurred over at least the 2-month period after 11 September) and blood collection at delivery, and given the estimated 3- to 4-month half-life of PAH–DNA adducts in white blood cells (Mooney et al. 2005), the measured adduct values may underestimate the effect of the event on the biomarker and reduce our ability to link exposure to developmental outcomes. Further, our sample size prevented us from examining whether outcomes differed depending on the week (or weeks) of exposure during pregnancy. Moreover, other unmeasured pollutants released by the WTC fires could have influenced MDI. Finally, we did not consider maternal distress during pregnancy which has been associated with decreased infant mental development (Huizink et al. 2003; Jones et al. 1998) and is a potential confounder or effect modifier.

## Conclusion

In conclusion, these results suggest that exposure to elevated levels of PAHs, indicated by PAH–DNA adducts in cord blood, may have contributed to a modest reduction in cognitive development among children prenatally exposed to the WTC event.

## Figures and Tables

**Table 1:** Characteristics of study subjects included in the developmental analysis compared with those not included.

Characteristic	Subjects included in the model (*n* = 98)	Subjects not included in the model (*n* = 105)^a^
Maternal age (years)	30.2 ± 5.2	29.8 ± 5.2
Household income (US$)^b^,^c^	24,564 ± 18,620	20,692 ± 16,597
Maternal education (%)^c^		
< High school	12.2	25.7
High school	17.4	17.1
> High school	70.4	57.1
Ethnicity/race (%)		
Asian	34.7	40.0
Black	13.3	14.3
White	40.8	40.0
Other/NA	11.2	5.7
Resided within 2 miles of the WTC in the month after 9/11 (%)^c^	34.7	19.0
No. (%) of nondetectable cord adducts	37 (37.8)	45 (42.9)
Cord adducts (adducts/10^8^ nucleotides)	0.24 ± 0.10	0.23 ± 0.10
Maternal exposure to ETS (% reporting at least one smoker in the home)^c^	24.5	11.4
Marital status (% married or living with a partner)	78.6	77.1
Material hardship (% reporting at least one unmet need such as going without or having inadequate food, housing, or clothing at some point in the past year)	10.2	6.7
Sex of newborns (% male)	50.0	41.9
Gestational duration (days)	276.6 ± 9.4	276.73 ± 9.4
Maternal intelligence (TONI)	97.4 ± 14.8	93.0 ± 13.9
3-year MDI	95.2 ± 11.9	94.4 ± 9.3^d^
3-year PDI^e^	98.1 ± 12.8	99.7 ± 13.3^f^

NA, not available. Values are mean ± SD except where noted.

a These 105 children had PAH–DNA adducts in cord blood but lacked MDI (*n* = 89) and/or demographic exposure data required for the analyses.

b Income based on midpoint of each of 10 household income categories, ranging from < $10,000 to > $90,000.

c *p* < 0.05, chi-square test.

d Only 16 subjects in this group had 3-year MDI test results.

e Four subjects were lacking PDI data, so *n* = 94.

f Only 15 subjects in this group had 3-year PDI test results.

**Table 2:** Results of multiple linear regression analyses of adducts (continuous), ETS, and 3-year MDI and PDI.^a^

	MDI (*n* = 98)		PDI (*n* = 94)^b^	
	β (95% CI)	*p*-Value	β (95% CI)	*p*-Value
Marital status^c^	5.51 (0.55 to 10.47)	0.03	1.32 (−5.30 to 7.94)	0.70
Material hardship^c^	−9.38 (−16.20 to −2.56)	0.01	−4.07 (−13.51 to 5.37)	0.40
TONI^c^	0.25 (0.11 to 0.39)	0.00	0.08 (−0.10 to 0.26)	0.37
ETS^c^	−2.25 (−6.90 to 2.40)	0.35	−0.58 (−7.07 to 5.91)	0.96
Cord adducts (Ln)^d^	4.18 (−0.95 to 9.31)	0.11	2.24 (−4.44 to 8.92)	0.51
Interaction of cord adducts and ETS	−12.25 (−22.66 to −1.84)	0.02	−0.07 (−14.85 to 14.71)	0.99

a Sex of newborns, ethnicity, maternal age, income, education, and gestational age were not included in the final model because their associations with MDI were not significant at the level of *p* < 0.1.

b The β is the regression coefficient. Four subjects did not have PDI results.

c Defined in Table 1.

d The log adduct variable is centered by subtracting the mean log cord adducts (−1.525) from each subject’s adduct measurement.
